# Left renal vein hypertension as a cause of occult hematuria: Multi-detector computed tomography demonstration

**DOI:** 10.4103/0970-1591.44274

**Published:** 2008

**Authors:** Nitin P. Ghonge, Bharat Aggarwal

**Affiliations:** Body Imaging Division, Department of Radio-Diagnosis, Diwan Chand Imaging Research Center, 10 B, KG Marg, New Delhi-110 001, India

## CASE REPORT

The illustrated case is a 55-year-old asymptomatic multiparous post-menopausal female who was diagnosed to have microscopic hematuria on serial urine cytology examinations [Phase contrast microscopy 3 100; mid-stream early-morning centrifuged specimens of urine]. There were 5-10 isomorphic erythrocytes per high-power field, without any proteinuria or pus cells in any examination. There was no history of diabetes, hypertension or any major illness in the past. The complete blood cell count, serum biochemistry and coagulation profile were within normal limits. Ultrasound examination did not reveal any definite structural abnormality in the urinary system. The multi-detector computed tomography examination, however, showed significant dilatation of the left ovarian vein with formation of pelvic varices in the broad ligament [Figure 1a and b]. There was Grade 3 venous reflux from the left renal vein (LRV) into the left ovarian vein and LRV ratio was found to be approximately 5 in this case [Figure 2a and b]. The right-sided pelvic varix in this case is secondary to contrast reflux through the intercommunicating anastomotic veins along the para-uterine venous plexus. The formation of pelvic varix on the right side and delineation of right ovarian vein suggest severity of venous reflux from LRV into left ovarian vein. No structural abnormality was detected in the urinary system. These set of images illustrates LRV hypertension as an unusual cause of occult hematuria which was diagnosed on MDCT. The patient was treated by conservative management and is currently under close follow-up, since the last six months. The role of invasive venography, therapeutic endovascular interventions and surgical options was explained to the patient. The last urine microscopy findings, however, remain unchanged.

**Figure 1a:**
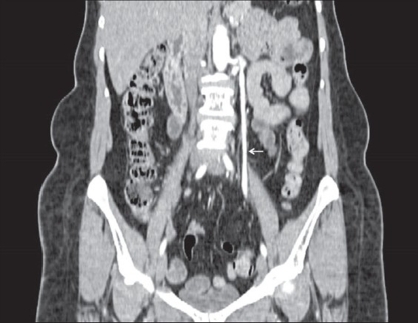
MDCT-coronal reformatted image shows significant dilatation of left ovarian vein (arrow). High-contrast density in the left ovarian vein is secondary to reflux of the contrast medium from the left renal vein into left ovarian vein

**Figure 1b:**
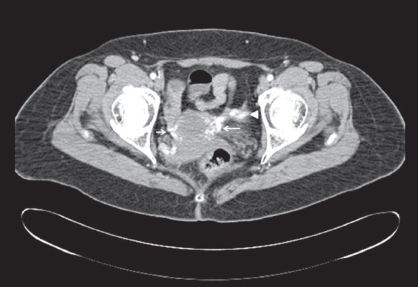
MDCT-axial image shows bilateral pelvic varices in broad ligament. The varix is larger on the left (long arrow) as compared to the right (short arrow) side. Dilated distal left ovarian vein is also noted (arrowhead)

**Figure 2a:**
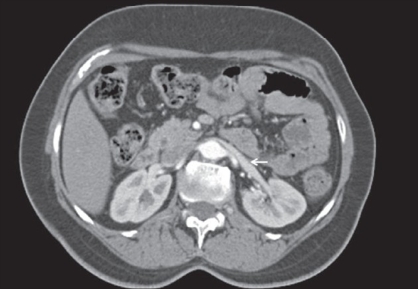
MDCT-axial image shows dilatation of left renal vein in the lateral segment, at the level of the left ovarian vein confluence (arrow)

**Figure 2b:**
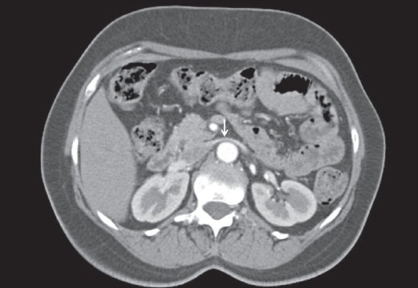
MDCT-axial image (cranial to figure 2a) shows narrowing of left renal vein in aorto-mesenteric segment (arrow). The altered dimensions of the left renal vein are quantified in terms of LRV ratio, which is raised in this case

## DISCUSSION

Nutcracker phenomenon was first described by de Schepper in 1972, as compression of the LRV, usually in the fork between the aorta and superior mesenteric artery.[1] The compression of the LRV may lead to venous hypertension and consequent formation of venous collateral channels in the renal hilum and peri-ureteric regions. There may be associated dilatation of the gonadal veins with clinically significant pelvic varices in the females and varicocele in males. This clinical entity has been termed as nutcracker syndrome (NS), while the asymptomatic dilatation of LRV is referred to as the nutcracker phenomenon. The precise etiologic factors are not known but several possible causes have been proposed. This includes ptosis of kidneys posteriorly, a retro-aortic position of LRV and an abnormally high course of LRV. These anatomical variations result in stretching of the LRV and its poor venous drainage.[2,3] The abnormal branching of the superior mesenteric artery [SMA] from the aorta is also supposed to be responsible for LRV compression, in a manner similar to the SMA syndrome. The latter refers to duodenal obstruction secondary to its compression between the aorta and SMA. On the basis of their findings on sagittal magnetic resonance imaging, Hohenfellner *et al*., suggest the abnormal angle of the origin and course of the proximal part of SMA to be responsible for this phenomenon.[4] The role of Doppler sonography in upright position is also described to evaluate the possibility of NS. The ultrasound done in upright position revealed comparatively narrower SMA-aorta ‘take-off’ angles, as compared to supine position.[5] Aberrant right renal artery is also described as a cause of LRV compression and symptomatic NS.[6]

Kim *et al.*, described the role of LRV diameter and peak flow velocity measurements on B-mode ultrasound and Doppler respectively.[7] The LRV can be anatomically categorized into the lateral segment and the medially-located aorto-mesenteric segment. The LRV diameter ratios and the velocity ratios exceeding 5.0 are suggested as threshold values for the diagnosis of NS. The sensitivity and specificity of velocity ratios were reported to be higher than those of diameter ratios (sensitivity 80% vs. 69%; specificity 94% vs. 89%) in the diagnosis of NS.[7] The B-mode ultrasound, however, did not reveal any definite structural abnormality in the urinary system in our case. The Doppler was not performed as the diagnosis of LRV hypertension was not suspected initially.

The patients with nutcracker phenomenon are usually young and thin. The nutcracker phenomenon may be an important cause of pediatric varicocele. In children, the role of Doppler US for the diagnosis of nutcracker phenomenon is affected by the fact that the LRV sampling area is smaller. Apart from this, the Doppler angle is larger in children than in adults. The high reno-caval pressure gradient may lead to retrograde blood flow from the LRV into the testicular vein. Doppler spectral analysis and venography of the LRV are useful in children with varicocele to look for the presence of underlying nutcracker phenomena.[8] Compression in the aorto-mesenteric segment of the LRV with dilatation of the lateral segment and formation of collateral venous routes are hallmarks of NS. The left renal vein hypertension may result in rupture of thin-walled veins into renal calyceal fornices and consequent microscopic or intermittent macroscopic hematuria with or without left flank pain.[9,10] The occult hematuria may present with anemia and proteinuria.[11] The ‘pelvic congestion syndrome’ is yet another clinical presentation of NS in adults and the patient usually presents with dysmenorrhea, dyspareunia, post-coital ache, lower abdominal pain, dysuria, pelvic, vulvar, gluteal or thigh varices and emotional disturbances.[12] As much as 88% of patients with left renal bleeding of unknown origin had LRV hypertension which can be confirmed with pressure gradients of more than 1-3 mm Hg between LRV and inferior vena cava, and leads to backflow in collateral veins, including the gonadal, ascending, adrenal, periureteral and capsular veins in invasive venography.[13]

The MDCT provides an important noninvasive alternative to diagnose this condition based on LRV dimensions, reflux of contrast medium from LRV into left ovarian vein and delineation of collaterals. The LRV dimensions are measured as antero-posterior dimension in the axial planes at two different points. The first measurement is done for the lateral segment of LRV at a point where the left ovarian vein drains into the LRV. The second measurement is done for the aorto-mesenteric segment of LRV at the narrowest point where the LRV passes between the aorta and the superior mesenteric artery. The dimensions are expressed as LRV ratio which is the ratio of measurements in the lateral segment and aorto-mesenteric segments of LRV.[14] Reflux into the left ovarian vein was defined as early opacification of the ovarian vein occurring simultaneously with opacification of the renal veins. The reflux into the ovarian veins is graded from 1 to 3. Grade 1 is the reflux confined to the left ovarian vein without formation of pelvic varix. Grade 2 is the reflux in the left ovarian vein with formation of pelvic varix on the left side only. In Grade 3, there is formation of the additional pelvic varix on the contralateral side with contrast reflux up to the right ovarian vein. The contrast reflux across the midline is facilitated by the presence of inter-communicating para-uterine venous plexuses. The reflux into the left ovarian vein and the associated para-uterine varices is often seen in asymptomatic multiparous women and may not always be pathological. Significant dilation of ovarian veins normally occurs during pregnancy, as a compensatory response which may be the cause of higher likelihood of left ovarian incompetence in multiparous women. The left ovarian vein reflux becomes significant if it is associated with altered LRV ratios. The incidence of reflux was found to be 75% with LRV ratios greater than 2.1.[14] The mean LRV diameter ratio in this study was 3.6, which is lower than the cutoff value of 5.0 for the diagnosis of symptomatic NS. This may provide partial explanation for the absence of symptoms in most of the cases in this study despite the presence of left ovarian vein reflux. The visualization of venous collaterals provides crucial evidence in favor of LRV hypertension which can also be detected on MDCT. In our case, there was Grade 3 venous reflux into left ovarian vein and the LRV ratio was found to be approximately 5. There were, however, no definite demonstrable collaterals on MDCT.

The treatment of NS significantly depends on the patient symptoms and urine microscopy findings. The patients with mild hematuria can be offered conservative treatment with close follow-up.[15] The illustrated case was also managed conservatively. Massive hematuria and pain may require pressure gradient measurements with invasive venography followed by surgery or endovascular treatment. The surgical options include medial nephropexy with excision of renal varicosities,[16] renal vein bypass,[17] transposition of LRV[18] and auto-transplantation of kidneys.[19] The minimally invasive endovascular treatment options include embolization of the gonadal vein and pelvic collaterals[20] and stenting of the LRV.[21,22]

In the absence of structural causes, LRV hypertension or ‘nutcracker syndrome’ should always be ruled out in a case of macroscopic or microscopic hematuria. The phase-contrast urine microscopy shows isomorphic erythrocytes. B-mode ultrasound including Doppler assessment and MDCT are the primary imaging modalities. Invasive venography may be required for further evaluation. It is, however, important to differentiate asymptomatic dilatation of LRV (nutcracker phenomenon) and ‘nutcracker syndrome’ presenting with gross or microscopic hematuria, orthostatic proteinuria, varicocele and hypertension. The diagnosis of LRV hypertension should be made with caution in multiparous women. As illustrated in the present case of ‘hematuria of unknown origin’, the MDCT diagnosis of LRV hypertension is feasible in clinical practice if due attention is paid to finer imaging details and the index of suspicion is high.
